# *Proteopedia *- a scientific 'wiki' bridging the rift between three-dimensional structure and function of biomacromolecules

**DOI:** 10.1186/gb-2008-9-8-r121

**Published:** 2008-08-03

**Authors:** Eran Hodis, Jaime Prilusky, Eric Martz, Israel Silman, John Moult, Joel L Sussman

**Affiliations:** 1 Department of Structural Biology, Weizmann Institute of Science, Rehovot 76100, Israel; 2 The Israel Structural Proteomics Center, Weizmann Institute of Science, Rehovot 76100, Israel; 3 Biological Services Unit, Weizmann Institute of Science, Rehovot 76100, Israel; 4 Department of Microbiology, University of Massachusetts, Amherst, MA 01003, USA; 5 Neurobiology Department, Weizmann Institute of Science, Rehovot 76100, Israel; 6 Center for Advanced Research in Biotechnology, University of Maryland Biotechnology Institute, Rockville, MD 20850, USA; 7 Current Address: Department of Computer Science and Applied Mathematics, Weizmann Institute of Science, Rehovot 76100, Israel

## Abstract

Many scientists lack the background to fully utilize the wealth of solved three-dimensional biomacromolecule structures. Thus, a resource is needed to present structure/function information in a user-friendly manner to a broad scientific audience. *Proteopedia * is an interactive, wiki web-resource whose pages have embedded three-dimensional structures surrounded by descriptive text containing hyperlinks that change the appearance (view, representations, colors, labels) of the adjacent three-dimensional structure to reflect the concept explained in the text.

## Abstract 2

Proteopedia is an interactive wiki-style web resource that presents 3D structural and functional information in a user-friendly manner and allows real-time community annotation.

## Rationale

Structural biology has played a central role in fueling the massive advances made by the life sciences in the last few decades. More than a dozen Nobel prizes have been awarded for achievements in structural biology since solution of the structure of the DNA double helix in the early 1950s was followed by solution of the first protein structures at the end of the same decade. Beautiful images of three-dimensional structures regularly adorn the covers of *Science*, *Nature *and *Cell*. Indeed, a wealth of protein structures has been solved in recent years, and entries in the Protein Data Bank (PDB) [1,2] now number over 50,000. But structural information is surprisingly still not in the mainstream of biology for the simple reason that three-dimensional structures are often hard to understand, even for a structural biologist. The widely held impression is that these structures are understood in detail and put to use in research; in fact, the structures are hardly discussed at all, especially by biologists lacking a structural background. While computer graphics software greatly aids in the understanding of these structures by displaying them in three-dimensions, the pages of printed scientific journals flatten the structures to a two-dimensional image, with much of the three-dimensional information thus being lost. It should be noted, however, that a number of journals (*Nature*, *Nature Structural and Molecular Biology*, *ACS Chemical Biology* and *Molecular Biosystems*) have begun to offer links to FirstGlance in Jmol [3] for interactive three-dimensional structure visualization, and two journals (*ACS Chemical Biology *and *Biochemical Journal*) occasionally offer interactive three-dimensional figures crafted by Molecules In Motion [4]; but these still lack the simple direct link between the printed information and the three-dimensional structures that is provided by *Proteopedia*. Moreover, many biologists have a limited knowledge of chemistry; thus, structural biologists need to make a special effort to develop tools that make macromolecular structures accessible and useful to the life science and clinical communities.

One such tool is molecular animation. Movies are successful at making biomacromolecules and their complexes come to life on the screen, and thus are often able to preserve and convey three-dimensional information far better than static two-dimensional images. Previous efforts to communicate the structural and functional features of a biomacromolecule have largely focused on creation of such movies and on interactive visualizations (for example, Kinemage [5], MovieMaker [6], Protein Explorer [7,8], Protein Movie Generator [9], and PDB2MGIF [10,11]). Until recently, the time and technical knowledge required to make such macromolecular animations were daunting. This has been partly rectified with the advent of eMovie [12], a plug-in for the molecular visualization program PyMOL [13], and PolyView3D [14,15], which have both simplified the creation process and lowered the threshold for sharing molecular three-dimensional information via movies. However, although movies are excellent for individual presentations, they are not an adequate solution to the problem that we are attempting to address, because they are fixed once created, and provide neither an interactive environment nor integration with textual information.

What is missing is a common resource that would make three-dimensional structures easier to understand, permit linking of function to structure, and at the same time simplify the sharing of structural information. This should be accomplished not by reducing the amount of information conveyed, but rather by making three-dimensional information intuitive, and thus more accessible to all. Already, valuable attempts have been made to tackle this problem. Perhaps the most notable recent example is iSee [16], which, like Kinemage, makes three-dimensional structures more intuitive by linking textual information to three-dimensional views of the structure. However, iSee uses both proprietary authoring tools, which must be purchased, and a proprietary viewer that has to be downloaded and installed in order to view both text and three-dimensional structures.

For non-structural biologists, the issue is not understanding a structure as an end in itself, but relating the structural information to biological applications: for example, how do mutations cause disease? Or, to be more specific, what mutation can be performed that will prevent one protein from interacting with another? How can one design a drug that will stabilize a protein destabilized by mutagenesis? Which part of a protein may be useful as an epitope? What happens in an organism in which a given protein domain is missing? In order for structural biology to provide genuine added value for non-structural biologists, we need a resource that will allow the relevant information and its analysis to be entered by the appropriate, knowledgeable scientists - and easily accessed and understood by users without a formal background in structural biology.

*Proteopedia *is a wiki-based web-resource that has been designed to address what is missing from structural biology: a mechanism for making three-dimensional structures easier to understand, a linking of function to interactive three-dimensional structure visualization, and a simplified sharing of structural and functional knowledge (a wiki is a resource or website where users can edit the pages in the website using simple text-editing tools). This resource is a tool for all scientists who need to utilize three-dimensional structural information in their research, as well as for educators requiring a medium for compelling presentation of structure-function relationships. *Proteopedia *is also meant for structural biology specialists in need of a more effective method of communicating their results. As a website, *Proteopedia *is freely accessible to all users without the need for downloading and installing any software. (Java is required. Most users will find that they already have Java installed on their computers. Should they need to download Java, they will be directed to the Java website for the free and simple download.). Furthermore, adding content to the website is simple: textual content is added in the same way as it is added in Wikipedia [17], taking advantage of an interface that is familiar to millions. Interactive, customized scenes of three-dimensional structures linked to the text are simple to add via *Proteopedia*'s easy-to-use *Scene Authoring Tools*. *Proteopedia *is intended to be the website of first-resort for everyone from research scientists to students seeking integrated three-dimensional structural and functional information about a particular protein or molecule.

**Figure 1:**
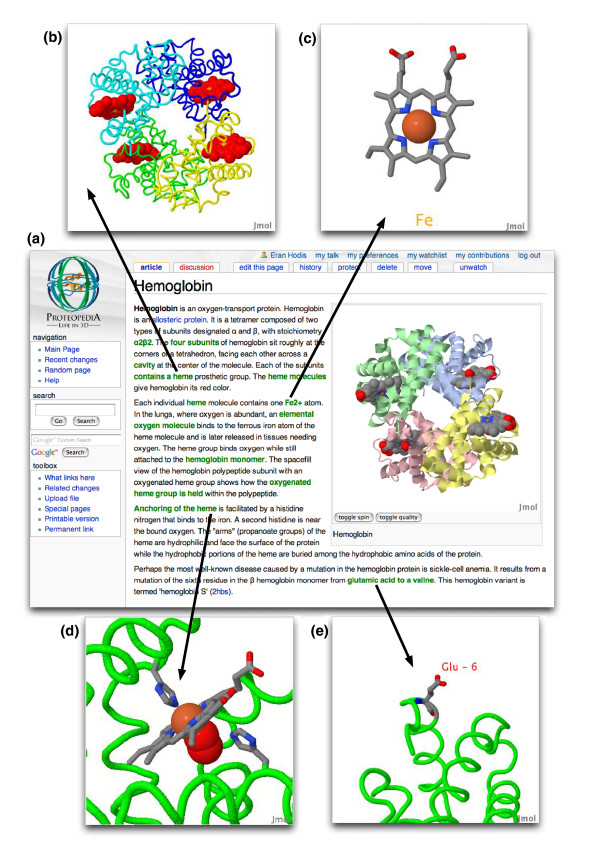
Green links change from one easily authored molecular scene to another. **(a) **For example, a user interested in hemoglobin visits the page of that name in *Proteopedia *(see [41]), which then loads with a slowly rotating crystal structure of hemoglobin in an interactive Jmol applet. **(b) **As the user reads that hemoglobin is a tetramer and that each of its subunits contains a heme prosthetic group, she or he can click on a green link in the corresponding text, eliciting a change in the hemoglobin in the Jmol applet, coloring each subunit a different color and displaying them in a smoothed trace of their α-carbon backbones, so that the hemes, colored in red, are easily visible. **(c, d) **While reading a sentence explaining that each heme contains an Fe^2+ ^atom and clicking the appropriate green link, the user can watch the virtual hemoglobin molecule slowly rotate to a viewpoint that displays only a single heme, zoomed in, with its Fe^2+ ^atom highlighted (c) or anchored to the protein (d). **(e) **When the user clicks on 'glutamic acid to a valine' he or she can see the specific point mutation in the hemoglobin molecule that causes sickle-cell anemia. Thus, text discussing and describing the structure and function is reinforced by immediate and specific three-dimensional visualization.

*Proteopedia *has three defining features. First, three-dimensional information is presented in an intuitive manner: descriptive text contains hyperlinks that change the adjacently displayed three-dimensional structures to coincide with points made in the text. (Figure 1). (The visualizations in *Proteopedia *are, in fact, not truly three-dimensional, but the impression of three-dimensionality is achieved by having the structure rotate, a visualization technique pioneered by Levinthal in the 1970s [18].) Second, there is no requirement for installation and operation of downloadable viewers. A web browser is all that is needed for full access, including both interactive three-dimensional viewing and content authoring. The site works equally well on Windows, Mac OS X, and Linux. Third, content can be easily added by any approved, knowledgeable user, via simple-to-use authoring tools.

## Proteopedia

### *Proteopedia *shows and tells

At first sight, *Proteopedia *looks a lot like Wikipedia. Indeed, *Proteopedia *runs on the same open software wiki package used by Wikipedia, MediaWiki [19]. However, a *Proteopedia *user will soon notice several differences. For one, most pages include at least one instance of the molecular visualization applet Jmol [20] (an applet is a small program embedded in a webpage), displaying a slowly revolving three-dimensional protein structure. Instead of a flattened, two-dimensional image of a protein structure, users are greeted by a three-dimensional structure that may be rotated and explored in real-time. The second most obvious difference is the existence of green hyperlinks within the text. Clicking on these hyperlinks changes the three-dimensional molecular scene displayed within the adjacent Jmol applet to one that better illustrates the concept referred to in the relevant text. In some sense this follows the familiar and important English essay-writing adage "Show, don't tell".

For example, a user interested in hemoglobin visits the page of that name in *Proteopedia*. A slowly rotating three-dimensional crystal structure of hemoglobin is displayed in an interactive Jmol applet. While reading the text, the user clicks on the embedded green hyperlinks to display new molecular scenes illustrating the points in the text (Figure 1). Each of the links, which can be traversed in any order, smoothly transitions from the previous scene to the next one, enhancing the user's spatial comprehension of relative locations on and within the protein. In contrast, two-dimensional images of protein structures often leave the user grappling with the spatial relations of one image to another.

### Creating molecular scenes without tears

The key breakthrough in *Proteopedia *is the ease with which any user can create 'text-to-molecular-scene links' using the *Scene Authoring Tools *(for example, see [21] for a narrated video tutorial). The *Scene Authoring Tools *strive for user-friendliness, and they can be accessed by virtually any system, be it Windows, Linux, or Mac, running any of the most popular web browsers (Internet Explorer, Firefox, Safari, and others).

A *Proteopedia *user who wants to create a scene uses the *Scene Authoring Tools *to manipulate his or her three-dimensional structure into the desired viewing-perspective and zoom, colors, representations and labels (like a two-dimensional picture). That particular scene of the three-dimensional structure is then saved and married to a green link in the text of the page. Whenever that green link is clicked, the Jmol applet will recall the saved scene, and will automatically transition smoothly to it. Conformational changes (or morphs) can be animated as well. Previously created scenes are easily recalled and edited within the *Scene Authoring Tools*.

### Content from the user community, wiki-style

Each page in *Proteopedia *can be modified by the members of the user community, thus permitting addition and editing of content. Modifications become visible and searchable immediately. Adding and editing content is quick, easy, and accessible to the common non-technical user and scientist.

Compared to other three-dimensional structural databases that solely archive, in a rigid format, data from scientists working on a given protein, *Proteopedia*, because it is a wiki, permits anyone knowledgeable with respect to that particular protein to add information regarding its function and to relate the information directly to the three-dimensional structure. Mistakes and errors are easily corrected by users who have opted to receive e-mail notification whenever the page on which they are expert is changed. Each change made to a page is logged in that page's history, so that pages can easily be reverted to a previous state. When appropriate or necessary, a page may be protected from being edited except by a selected group of stewards who can evaluate proposed changes to the page.

### Adaptation of the wiki concept for the scientific community

In creating a wiki for the scientific community, two chief concerns are to ensure that only knowledgeable users are authoring content, and to ensure that authors receive proper credit for their contributions. *Proteopedia *addresses these issues in the following manner. While anyone can view *Proteopedia *pages, only registered users can edit pages and add content. In contrast to Wikipedia, *Proteopedia *user accounts are exclusive to the scientific community, and only scientists, educators, and students of science are invited to request accounts by clicking on "log in/request account" at the upper right-hand side of the webpage. Approved accounts are created using the users' real names so that the authors both receive appropriate credit for their contributions (each page lists the names of the people who have contributed to the page) and take responsibility for their entries.

### *Proteopedia *for lectures and for supplementing journal articles: protected pages

In a departure from the purist wiki model, *Proteopedia *provides each user with a section where she or he can create pages that are protected from editing by others. By so doing, *Proteopedia *encourages educators and lecturers to take advantage of the three-dimensional visualization features of *Proteopedia *to create interactive three-dimensional 'lecture slides' for projection from the website, without having to worry that the content might be changed by someone else. Students can access this lecture material at any time, anywhere, even after the lecture. Additionally, scientific papers discussing three-dimensional macromolecular structures may also benefit from the three-dimensional visualization features of *Proteopedia *via protected pages with interactive, three-dimensional material supplementary to the publication.

### 50,000 pages and growing

**Figure 2:**
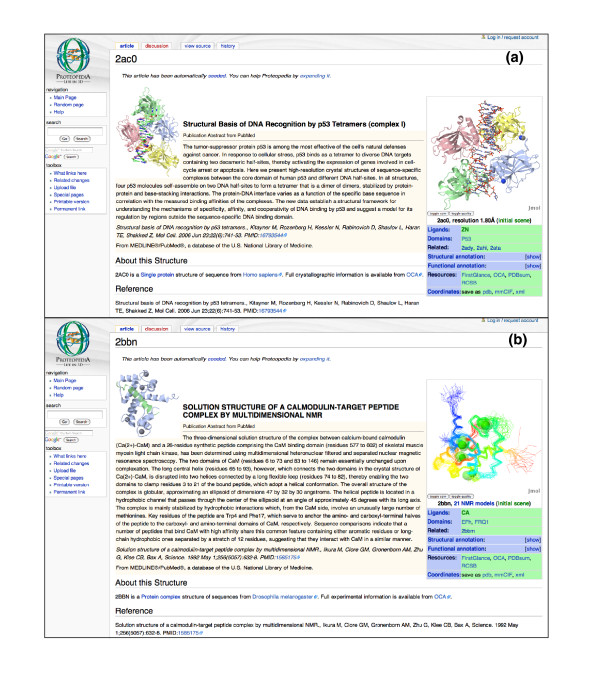
Automatically seeded pages for each of the over 50,000 entries in the PDB. **(a) **The *Proteopedia *page '2ac0' [42] contains the structure of a protein-DNA complex showing the structural basis of DNA recognition by P53 tetramers [43]. **(b) **The *Proteopedia *page '2bbn' [44] shows an ensemble of 21 NMR (nuclear magnetic resonance) models for the solution structure of a calmodulin-target peptide complex [45]. Note how the ensemble of the 21 NMR models reveals the more flexible portions of the protein structure. These are two examples (a, b) of automatically seeded pages created for PDB entries. The title sentence, in bold, comes from the title in the PDB file, and the "Published Abstract from PubMed" section text comes from the abstract of the article associated with the structure (retrieved from PubMed). A rotatable/zoomable three-dimensional structure in Jmol is displayed, and, under it, useful information about the structure including green scene links highlighting functional sites and ligands (as available in the PDB file), plus a link for further visual exploration in FirstGlance in Jmol, and links to related genes, domains, related PDB entries, structural annotation (InterPro, Pfam, UniProt, SCOP, CATH), functional annotation (GO and GeneCards), links to other resources, and links to download the coordinates of the structure. The side-by-side placement of the abstract and the three-dimensional structure is of immediate value, and these seeded pages also serve as strong starting points for addition of content. The amount of information available on each PDB entry varies, and thus so do the lengths of seeded pages.

*Proteopedia *is already online, serving the scientific community. It contains automatically seeded pages for each of the more than 50,000 entries in the PDB, updated weekly with each release of new PDB entries. Each such page includes, along with a rotatable/zoomable three-dimensional structure, the abstract of the paper associated with the structure (from PubMed [22]), green hyperlinks that highlight key parts of the structure defined in the PDB file (for example, ligands and functional sites) and other useful information detailed in Figure 2. A user familiar with a structure will thus find its page ripe and ready for enhancement with additional content and new scenes to better illustrate the function of the protein - much easier than starting from a blank page. Additionally, these PDB entry seed pages have high value to a diverse audience of scientists even before insertion of user-added content due to the inherent convenience of having an interactive, three-dimensional visualization of the structure adjacent to the abstract of its publication.

But *Proteopedia *is not a one-to-one mapping of the PDB. The seeded PDB entry pages in *Proteopedia *provide a base level in a hierarchical organization. A higher level consists of pages that explain and summarize structure/function knowledge about particular molecules or classes of molecules. For example, the hemoglobin and acetylcholinesterase pages provide general overviews of these molecules along with rotatable/zoomable three-dimensional structures and links to all of the related PDB entry pages in *Proteopedia*.

### If you build it, they will come

To have real value to a diverse audience, three-dimensional structures of proteins, RNA, DNA, and other biomacromolecules must be communicated, wherever possible, together with their biochemical and biological functions. While *Proteopedia *makes this integrated communication possible, and even simple, it is a resource that relies on community-annotation, and there is no guarantee that enough knowledgeable users will take to *Proteopedia en masse *to reach a critical level of users. To minimize this risk, *Proteopedia *attempts to be as enticing as possible to these knowledgeable users, with intuitive visualization features, with user-friendly authoring tools, with attribution of content, with special protected pages for lectures, tutorials, and supplementary information for journal articles, and with a familiar interface (from Wikipedia). In addition, all textual content and scenes added by users to *Proteopedia *are licensed under the GNU Free Documentation License (as in Wikipedia), thus ensuring that the content is free, and that *Proteopedia *is solely a vehicle for content creation and dissemination. *Proteopedia *will also continue to cater to its knowledgeable users by listening to their feedback and actively developing in ways that satisfy their needs and desires. For example, *Proteopedia *will shortly offer the option to display the amino acids in three-dimensional protein structures color-coded according to their degree of evolutionary conservation (using ConSurf [23]).

### How *Proteopedia *is being used today

**Figure 3:**
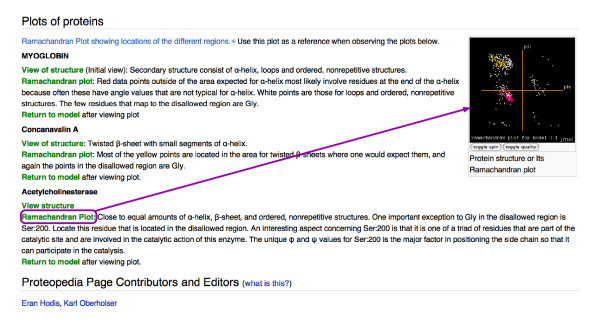
An example of a protected page: a tutorial on Ramachandran plots. This image shows a section of a page containing a tutorial on Ramachandran plots. The green links in this section allow the user reading the page to compare the Ramachandran plots of three proteins with dissimilar structures by first viewing the three-dimensional structure of a particular protein and then viewing its Ramachandran plot. The plot displayed in the Jmol applet in the figure is that of acetylcholinesterase from PDB file 1eve. Points on the plot representing residues from α-helices are drawn in red, points representing residues from β-sheets are yellow, and points representing the other residues are in white. This page was authored by Professor Karl Oberholser, Department of Chemistry and Biochemistry, Messiah College, PA, USA. The page is titled "User:Karl_Oberholser/Ramachandran_Plots" (see [46]). "User:Karl_Oberholser" is Karl Oberholser's userpage, and this Ramachandran Plot page is a subpage of his userpage. All userpages and subpages thereof are editable only by their eponymous users. Since this Ramachandran plot page is a subpage of a userpage, it is editable only by Karl Oberholser, and is referred to as a protected page. He can count on this protected page in the wiki being unchanged, and use it as a three-dimensional "lecture slide". Since all user-added content in *Proteopedia *is released under the GNU FDL, other users may copy content from this page and add it to a publicly editable page, or another protected page, in *Proteopedia *with proper attribution to its author.

The number of user-created and user-enhanced pages currently number in the double digits. User added content is expected to rise following publication of this paper, but over 100 users have already registered accounts. These *Proteopedia *users have started to develop several protein and molecule pages (see, for example, [24], a page on recoverin, a calcium-activated myristoyl switch), and have also expanded the seeded pages for the PDB entries they have authored or know well (see, for example, [25], a page on PDB entry 2rkx from a recent, exciting study of an enzyme designed for a reaction not catalyzed by a naturally occurring biocatalyst [26]). In one case, *Proteopedia *was used to render in three-dimensions several figures from a publication before a journal club meeting (see [27], a page on the structure of a human p110alpha/p85alpha complex [28]). In another case, an undergraduate student created a page on Photosystem II in *Proteopedia *for a biochemistry class assignment (see [29]). Using the protected pages format, a university professor and educator has created a graphical tutorial on Ramachandran plots (Figure 3). A page on the highest impact structures of all time currently lists the DNA double helix (B form), myoglobin, lysozyme, deoxy-hemoglobin, transfer RNA, tobacco bushy stunt virus, major histocompatibility complex class I, and the ribosome, and invites contribution and discussion (see [30]).

### Key advantages of *Proteopedia*

*Proteopedia *is a novel resource, and its key advantages are as follows. First, it links text with interactive three-dimensional scenes of molecules and molecular complexes. Second, the three-dimensional scenes of molecules and molecular complexes can be created easily by *Proteopedia *users, using the *Proteopedia Scene Authoring Tools*, and immediately shared with and viewed by all. Third, it can be viewed via any standard browser and operating system, and requires no proprietary or commercial software. Fourth, in contrast with most other wikis, there are different levels of editorial control and input access, ranging from pages to which any registered user can contribute to protected pages, intended, for example, for teaching, which can be modified by only one author. Fifth, also in contrast with most other wikis, each page shows the full real names of its authors; thus, authors not only receive appropriate credit but also take responsibility for their contributions. Sixth, it features automated seeding of pages for each of the entries in the PDB, but with substantial added content. The added content includes the published abstract of the article associated with the structure, an interactive three-dimensional structure of the macromolecule with, where possible, links to key structural features, including the active site, ligands, and links to other relevant databases. These seeded pages provide valuable templates to which knowledgeable users can add content. Seventh, it extends beyond the contents of the PDB, providing for hierarchical organization of structure and function categories such as protein families, structural classes and biological function. Eighth, content is not restricted to PDB structures. Contributors can upload their own coordinates, experimental or theoretical models, whether of single biomolecules or of complexes. Theoretical models are clearly distinguished as such. Contributors may also add small molecules that are biologically relevant or that could benefit from *Proteopedia*'s visualization technology. Ninth, visualization is not restricted to a single format. Several are already incorporated, including Jmol, kinemages (using MageJava [31]), movies, morphs (for example conformational changes or docking actions), and images. Table 1 shows the unique combination of features in *Proteopedia *in comparison to related software tools.

**Table 1:** Unique features of *Proteopedia *in comparison to existing resources with similar purposes

Resource	Purpose	Contents (April 2008)	Web resource	Contains all entries in the PDB, updated automatically	Community annotation	Interactive three-dimensional within site with molecular scenes linked to text	User-friendly three-dimensional authoring tools, freely available
*Proteopedia*	A free, collaborative, three-dimensional encyclopedia of proteins and other molecules	One page for every PDB entry with abstract and interactive three-dimensional views, including functional sites and ligands (> 50,000 pages), plus several dozen well-developed higher-level pages (such as hemoglobin)	**Yes**	**Yes**	**Yes**	**Yes**	**Yes**
iSee	To communicate the results of the SGC and ideally of other groups that purchase the software	Results of the Structural Genomics Consortium (about 400 datapacks available)	No*	No	No^†^	**Yes**	No^‡^
Kinemage	To communicate scientific illustrations as interactive computer displays	Estimated to be in the thousands for a wide variety of proteins and biomacromolecules, and created by a diverse group of authors	No*	No	No^†^	**Yes**	**Yes**
TOPSAN	An annotation platform limited to the targets of the Protein Structure Initiative	Small subset of structural genomics results (< 2,000 pages)	**Yes**	No	**Yes**^§^	No	No
PDBWiki	A community annotated knowledge base of biological molecular structures	One-to-one mapping of the PDB with additional links and images (> 50,000 pages)	**Yes**	**Yes**	**Yes**	No	No

This table is limited to publicly accessible web resources for information on protein and macromolecules that emphasize macromolecular three-dimensional structure and permit community annotation. The requirement for permitting community annotation excludes resources such as OCA, PDB, JenaLib, and PDBSum. The requirement to emphasize macromolecular three-dimensional structure excludes resources such as Wikipedia. (see the resource websites [39,47-50]). *The option to display datapacks (called kinemages in the case of Kinemage) on the web exists, but no web resource exists with pages displaying each of the datapacks. ^†^Using the authoring tools, users may create new datapacks. In this sense, the datapacks available on the web are community annotated. However, datapacks do not evolve via expert community annotation like a wiki. ^‡^The authoring tools are commercially available. ^§^Most of the content of TOPSAN pages is fixed, but users can add/edit a Protein Summary section and add comments.

## Conclusion

Protein structures are not ends in themselves. Structural information must be placed in the appropriate biological context in order to be useful. To borrow from Greg Petsko, "Structures have value when they are part of a larger effort to understand the biochemical and biological functions of the protein in question... [Structure determination] is not the end in itself, nor should it be, not anymore..." [32]. Structures have value to a more diverse audience when three-dimensional structural information is smoothly integrated with biochemical and biological information. For example, it would be ideal if each new deposition in the PDB were accompanied by a well-developed page in *Proteopedia *by its authors, serving at least as a sort of 'News and Views', and touching on deeper details about the structure as necessary.

*Proteopedia *enhances the scientific community's ability to communicate complex three-dimensional information. Its integrated text and graphics allow for structural information to be conveyed in a manner that is accessible to a broad repertoire of scientists. Relevance of structure to function can be transmitted in a transparent fashion, and shared via simple tools for contributing to the website. Furthermore, *Proteopedia *has the capacity to leverage the resources of many diverse experts in varied fields rather than just the curators at a database site - and the ability to do so in an exciting, new medium.

## Implementation

*Proteopedia *is built upon a customized version of the MediaWiki [19] open software package, and integrates the Jmol [20] open-source Java applet viewer for chemical structures in three-dimensions using an adapted version of the Jmol MediaWiki Extension [33] with novel *Scene Authoring Tools *built specifically for *Proteopedia*. Kinemages are visualized in *Proteopedia *using MageJava [31]. PDB entry pages are automatically seeded using a script driven by OCA [34] (the browser/database for protein structure/function), which aggregates information from various resources (listed at [35]). SGKB[36] annotation plays a key part in OCA's data collection for seeding the PDB entry pages, and two-dimensional images for these pages are seeded from the RCSB PDB [37] and the Jena Library [38]. *Proteopedia *is backed up daily to both local and remote locations at the Weizmann Institute of Science, with incremental backups daily and full backups weekly.

## Abbreviations

PDB, Protein Data Bank.

## Authors' contributions

EH translated JLS's vision of an easy-to-use and universally accessible resource for communicating complex biological structural information into the first working version of *Proteopedia*, which included the *Proteopedia Scene Authoring Tools*. JP migrated this first working version of *Proteopedia *to an externally accessible server and developed and seeded the automatically created pages for each of the entries in the PDB as well as implemented several new and crucial features such as content attribution. EH and JP are active co-developers of *Proteopedia*. EM contributed to policy development, lent expert opinion, contributed content, and occasionally code, to the project. EM and JLS have been involved in testing and have provided ideas for new features, improvement of existing features, and for the project in general. IS and JM contributed expert opinion and guidance to the overall direction of the project. The idea for a resource like *Proteopedia *arose out of discussions between JM, JP, IS and JLS on the urgent need for better tools to integrate three-dimensional structure with functional information. JLS provided the main scientific and strategic guidance for the project. The manuscript was drafted by EH and all authors contributed revisions with JLS leading and coordinating the effort.
